# Does caring for grandchildren influence life satisfaction? A national-level analysis among older adults in India using a propensity score matching approach

**DOI:** 10.1186/s12889-025-23524-3

**Published:** 2025-07-04

**Authors:** Anukul Barman, Mihir Adhikary, Sunil Sarode

**Affiliations:** 1 Department of Population and Development, International Institute for Population Sciences, Mumbai, India; 2 Department of Public Health and Mortality Studies, International Institute for Population Sciences, Mumbai, India

**Keywords:** Grandchild care, Older adults, Life satisfaction, LASI, India

## Abstract

**Background:**

In the Indian socio-cultural context, the involvement of older adults in grandchild care is a normative expectation and a common household practice. Despite its prevalence, limited empirical evidence exists on how such caregiving responsibilities affect the psychological well-being and life satisfaction of older individuals. This study addresses this gap by examining the association between grandchild caregiving and life satisfaction among older adults in India.

**Method:**

This study draws upon nationally representative data from Wave 1 (2017–2018) of the Longitudinal Ageing Study in India (LASI), comprising 25,650 individuals aged 60 to 80 years. To examine the association between grandchild caregiving and life satisfaction among older adults, we employed Ordered Logistic Regression models to account for the ordinal nature of the outcome variable. Propensity Score Matching (PSM) was also applied to address potential selection bias and ensure comparability between caregivers and non-caregivers based on observed covariates.

**Results:**

The analysis revealed a statistically significant positive association between grandchild caregiving and life satisfaction among older adults. Specifically, individuals engaged in grandchild care exhibited higher odds of reporting greater life satisfaction (OR = 1.16; 95% CI: 1.10–1.23). Findings from the PSM analysis further supported this relationship, with an average treatment effect (ATE) of 0.057, indicating that caregiving for grandchildren contributes meaningfully to enhanced life satisfaction in later life.

**Conclusion:**

These findings underscore the positive role of grandchild caregiving in enhancing life satisfaction among older adults in India. Promoting supportive intergenerational relationships may therefore serve as an important strategy for improving psychological well-being in later life.

## Introduction

In many Asian societies, grandparental caregiving is a culturally embedded practice shaped by familial obligations and intergenerational reciprocity [16]. This phenomenon holds particular significance in India, as a substantial proportion of older adults reside in multigenerational households [14]. In such familial settings, grandparents often play a crucial role in caregiving grandchildren, alleviating their adult children's childcare burden, and supporting their offspring’s educational and economic advancement [3]. In return, grandparents may receive financial and emotional support, thereby enhancing their own economic stability and life satisfaction [29].

Life satisfaction represents a significant component of subjective well-being and constitutes a cognitive evaluative judgment regarding the quality of one's life [6, 7]. It is the extent to which a person positively assesses the overall quality of their life as a whole [24]. Consequently, it constitutes a comprehensive evaluation of an individual's quality of life based on his selected criteria and a significant catalyst for successful aging [26, 31]. Grandparenting has been shown to influence life satisfaction directly by fostering a sense of purpose and indirectly by reducing feelings of loneliness and improving self-esteem [30]. As a meaningful engagement, caregiving contributes to positive perceptions of aging, enhances intergenerational solidarity, and facilitates social integration, which is essential for improving older individuals' quality of life. Promoting such socially productive roles among older adults can contribute to building an age-friendly society [4]. Furthermore, cross-national studies have shown that cultural contexts shape the relationship between intergenerational caregiving and life satisfaction. In countries where caregiving is culturally expected, non-participation in such roles is often associated with lower life satisfaction [2].

However, existing research presents divergent findings regarding the impact of grandchild caregiving on older adults' well-being and life satisfaction. A considerable body of literature reports that caregiving can have positive effects, including enhanced life satisfaction, improved subjective well-being, better overall health, and reduced levels of depression [2, 4, 30, 33]. These outcomes are often explained through the lens of role enhancement theory, which posits that fulfilling multiple meaningful social roles can foster a sense of purpose, personal growth, and increased social integration [27]. Through active engagement in caregiving, older adults may experience emotional gratification, strengthened intergenerational bonds, and a reinforced sense of value within the family.

Conversely, other studies highlight the potential negative consequences of caregiving, including increased psychological burden, higher levels of depression, and reduced life satisfaction [5, 9, 18, 34].These adverse outcomes are consistent with role strain theory, which argues that occupying multiple demanding roles or encountering conflicting role expectations can lead to stress, emotional exhaustion, and compromised well-being [11]. Such strain may be exacerbated when caregiving is undertaken out of necessity, without adequate support or respite. These contrasting perspectives underscore the complexity of grandparental caregiving, suggesting that its impact on older adults' life satisfaction is highly context-dependent. It is shaped by caregiving intensity, the voluntariness of the role, cultural norms, and the availability of social and familial support.

Within the Indian context, the meaning and impact of caregiving are deeply intertwined with cultural values and social norms. Older adults often derive a sense of respect, purpose, and belonging through their involvement in grandchild care, reinforcing their integration within the family unit and safeguarding against post-retirement isolation and neglect [1]. Thus, caregiving in India is not merely a functional role but also a source of emotional and social significance.

Despite its importance, research on the relationship between grandchild caregiving and life satisfaction among Indian older adults remains relatively limited. While existing studies have explored broader determinants of life satisfaction—such as health status, living arrangements, social relationships, and economic conditions—less attention has been directed toward intergenerational caregiving as a potential contributor to subjective well-being [1, 34]. The present study investigates the association between grandchild caregiving and life satisfaction among older adults in India using data from the Longitudinal Ageing Study in India (LASI) to address this gap.

## Materials and methods

### Data source

The present study utilized data from the Longitudinal Ageing Study in India (LASI) – Wave 1 (2017–2018). LASI comprehensively collected data on various domains, including demographic characteristics, ageing, economic aspects, health status, biomarkers, health insurance, healthcare utilization, social relationships, social support, work and employment, retirement, and life satisfaction among older individuals in India [12]. It encompassed a substantial sample size of 73,396 older adults aged 45 and above, representing all states and union territories of India. LASI employed a rigorous multistage stratified area probability cluster sampling design to ensure the robustness and representativeness of the data. A three-stage sampling design was implemented in rural areas, while a four-stage sampling design was adopted in urban areas. The comprehensive methodology, detailing the survey design and data collection process, was meticulously documented and published in the report [12].

### Sample selection

**Fig. 1:**
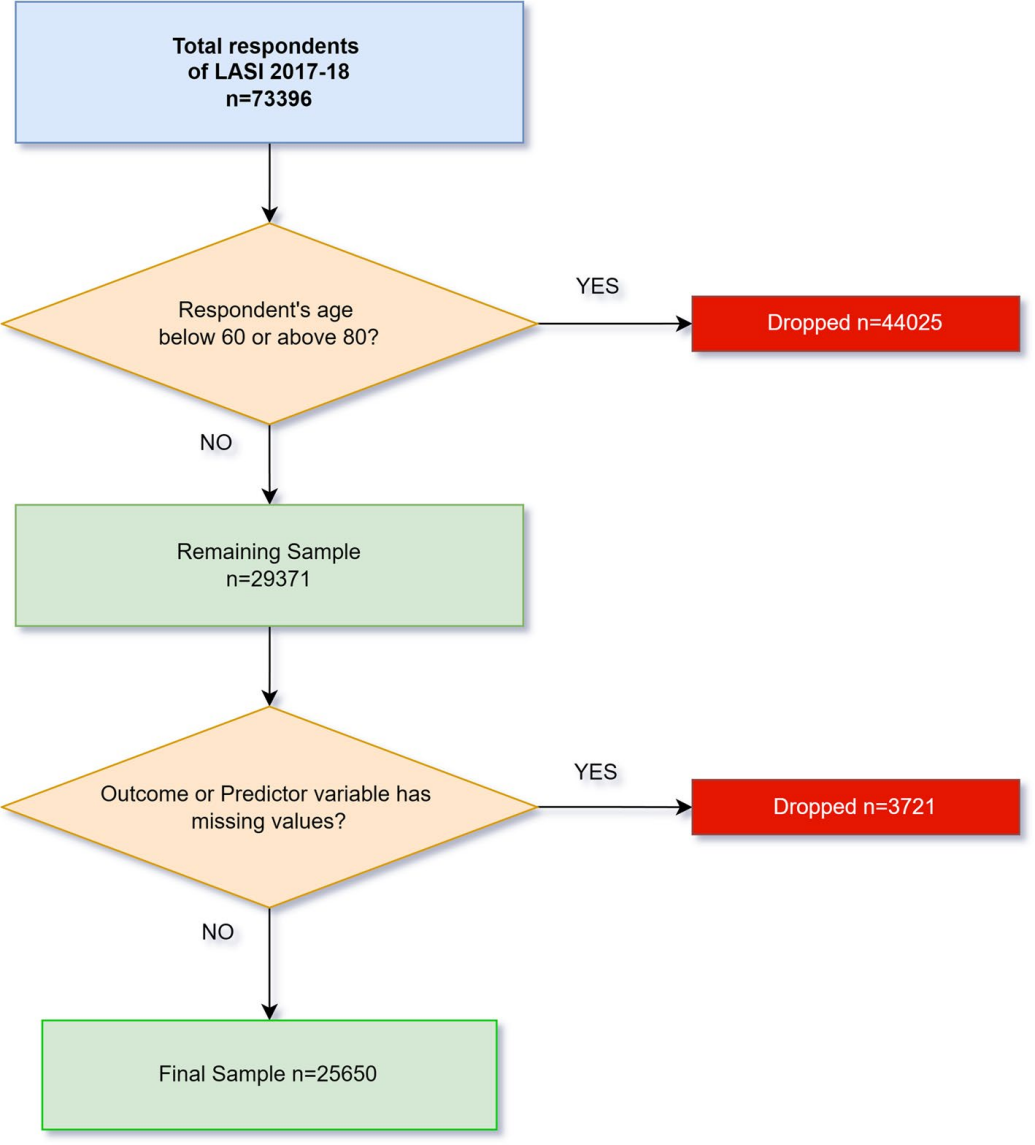
Data-cleaning process

Based on the research question's relevance, the study focused on older adults aged 60–80 years as the study population. This age range was chosen based on two primary considerations. Firstly, individuals over 80 years often do not take on intergenerational care due to factors such as their health status and the maturity of their grandchildren. Secondly, those over 80 years old may exhibit lower life satisfaction because of their health, and their inclusion may lead us to overestimate the correlation between intergenerational caregiving and life satisfaction. Therefore, to maintain the integrity and relevance of the study findings, samples over 80 years old were excluded. Furthermore, samples with missing or rejected responses on key variables were also removed. As a result, the valid sample size for this study was determined to be 25,650 individuals aged between 60 and 80 years. The detailed data-cleaning process is outlined in Fig. 1.

### Variable description

#### Dependent variable

The study's dependent variable is Life satisfaction, which was assessed using the Satisfaction with Life Scale (SWLS). The scale was designed to measure life satisfaction as a cognitive-judgemental process. Widely recognized and utilized, the SWLS is a robust measure of the life satisfaction component of subjective well-being [7].

SWLS comprises just five straightforward statements: “(I) In most ways, my life is close to ideal; (ii) the conditions of my life are excellent; (iii) I am satisfied with my life; (iv) so far, I have got the important things I want in life; and (v) if I could live my life again, I would change almost nothing”. A scale was assigned using a 7-point Likert scale across five statements, ranging from 1 (strongly disagree) to 7 (strongly agree). The total possible score ranges from 5 to 35. Scores between 5 and 9 indicate that the respondent is extremely dissatisfied with life, 10 to 14 reflect somewhat dissatisfied, and 15 to 19 indicate slightly dissatisfied. A score of 20 represents a neutral evaluation, scores from 21 to 25 indicate a slightly satisfied outlook, 26 to 30 reflect somewhat satisfied, and scores between 31 and 35 indicate the respondent is extremely satisfied with life [7, 22].

However, to simplify computation and interpretation, the scale is further categorized into three distinct categories: ‘low satisfaction’ (score of 5–20), ‘medium satisfaction’ (score of 21–25), and ‘high satisfaction’ (score of 26–35). The outcome variables were coded as 0 'low',1 'medium', and 2 'high'. This categorization was done based on existing literature [13, 15, 28].

#### Predictor variable

The key predictor variable in the present study is "Grandchild care", assessed through the question:"Do you look after any of these grandchildren?". Respondents were given two options: 'Yes' and 'No’. Accordingly, a binary variable was created, assigning a value of 1 to those who reported caring for their grandchildren and 0 to those who did not.

### Covariates

#### Socio-demographic covariates

The socio-economic covariates included in the analysis are as follows. The age of the respondents is categorized into two groups: 60–69 years (termed as “old”) and 70–80 years (referred to as “old-old”). Sex is categorized as male and female. Marital status is classified into “currently in the union” for individuals who were married or in a live-in relationship and “currently not in the union” for those who were widowed, divorced, separated, deserted, or never married. Living arrangements are categorized as living alone, living with spouse and/or others, living with spouse and children, living with children and others, and living with others only. Caste is classified into four groups: Scheduled Caste (SC), Scheduled Tribe (ST), Other Backward Class (OBC), and Others. Religion is categorized as Hindu, Muslim, Christian, and Other. Years of schooling are divided into four categories: 0 years, 1–5 years, 6–9 years, and 10 or more years of education. The place of residence is grouped into rural and urban. Social participation is categorized into a binary variable indicating “having any participation” or “not having any participation”. Finally, the region is divided into North, Central, East, Northeast, West, and South. The monthly per capita consumption expenditure (MPCE) quintile is derived from detailed household consumption data, including responses to eleven food-related and 29 non-food-related items. Food expenditure is assessed over a seven-day reference period, while non-food expenditure was captured over both 30-day and 365-day periods. To standardize reporting, MPCE is calculated using a uniform 30-day reference period and categorized into five quintiles, from the poorest to the richest households. Current work status is categorized as “currently working” and “currently not working.”

#### Health-related covariates

The health-related indicators include self-rated health, which is clubbed into two categories: 'poor' and 'good’. In assessing physical health, this study incorporated Instrumental Activities of Daily Living (IADL) [20]. IADL include tasks like cooking and serving a hot meal, buying groceries, making phone calls, managing medications, performing housework or gardening, handling finances, and navigating unfamiliar locations. IADL difficulties are categorized into "No limitation" for those who can perform all tasks independently and "Having limitations" for those who struggle with any [19, 21]. Lastly, as a mental health indicator, depression was included and was categorized into two categories- depressed (coded as 1) and not depressed (coded as 0). The depression scale employed in this study utilized the Short Form Composite International Diagnostic Interview (CIDI-SF), assessing psychological well-being with scores ranging from 0 to 10. Comprising ten questions with three or more positive responses assigned to “diagnosed with depression” [17].

### Statistical analysis

In this study, a diverse array of methods was employed to precisely ascertain the association between grandchild care and the life satisfaction of older adults. Firstly, using descriptive statistics, we presented a concise overview of the characteristics of the sample data. This includes demographic information such as age distribution, gender representation, socio-economic status, health status, and any relevant contextual variables. Secondly, we computed the prevalence of life satisfaction across various socio-demographic and health-related variables and assessed the significance of differences using chi-square tests. Thirdly, we conducted an ordered logistic regression analysis to see the association between grandchild caregiving and older adults' life satisfaction. Finally, to enhance the robustness and establish causality in the relationship between caregiving for grandchildren and life satisfaction, we employed propensity score matching (PSM) technique. It is a statistical technique used to reduce selection bias in observational studies by matching treated and untreated groups based on similar characteristics (propensity scores), making them more comparable for causal analysis.

Caregiving among older adults is not random but is shaped by various socio-demographic and personal factors. This suggests a self-selection effect in grandchild care, where differences in life satisfaction between caregivers and non-caregivers may stem more from the characteristics influencing the decision to provide care than from the caregiving role itself [8]. For instance, in the United States, intergenerational caregivers are frequently identified within lower socio-economic status groups [10]. This suggests that the effects attributed to intergenerational care may result more from selection biases than the caregiving itself. Hence, obtaining unbiased estimates solely through Order logistic regression poses challenges. To address the non-random nature of grandchild care behavior, we employed the PSM [23]. The basic principle of PSM is to match individuals in the treatment group with individuals in the control group who are as similar as possible based on the probability of obtaining a treatment, thus achieving a randomized treatment.

This study's treatment group comprised grandchild caregivers, and the control group consisted of non-careers. The specific steps were as follows. The initial step involves employing the logit regression model to compute propensity scores based on observable characteristics using the following formula-

$$PS\left(X\right)=\text{Pr}\left\{D=1\right|X\}=E\{D|X\}$$

Where $D$ is the dummy variable for whether the older adults was a caregiver. If the older adults was a caregiver then $D=1$, otherwise$D=0$. $X$ represents the covariates that effect whether an older adult is a caregiver.

Subsequently, the treatment group is matched with the control group based on these propensity scores. In this study, we adopted nearest-neighbor matching without replacement as the matching method. Finally, the treatment effect on the treatment group is determined by analyzing the matched data using the formula-

$$ATT=E\left({satisfaction}_{1}\right|D=1)-E\left({satisfaction}_{0}\right|D=0)$$

where, $satisfaction_1$ represents the life satisfaction of the caregivers; $satisfaction_0$ represents the life satisfaction of the non-caregivers; and $ATT$ is the difference between life satisfaction of the caregivers and life satisfaction of the non-caregivers.

## Results

### Characteristics of the study sample

Table 1 represents the descriptive statistics of the study population. The total observations in this study comprise 25,650. Most of the respondents (64.49%) were in the 60–69 age group, while 35.51% were aged 70–80 years. Females represented a slightly higher proportion (53.56%) than males (46.44%). Most participants were currently in a marital union (66.72%), whereas 33.28% were not. Regarding grandchild care, 19.6% of respondents reported caregiving involvement. Regarding living arrangements, the largest share (43.13%) lived with their spouse and children, followed by those living with children and others (27.12%). A small proportion lived alone (5.08%) or with others only (3.90%). Caste-wise, 38.76% belonged to the Other Backward Class, 25.21% to other general castes, 16.93% to Scheduled Castes, and 15.94% to Scheduled Tribes. Educationally, over half (54.17%) of the older adults had no formal schooling, while only 14.13% had 10 or more years of education. Regarding work status, 40.54% were not currently working, whereas 31.03% reported being engaged. Most resided in rural areas (67.24%), and the rest (32.76%) were urban dwellers. Regionally, the highest representation came from the southern region (27.81%), followed by the northern (18.81%) and eastern (17.11%) regions. Religiously, most participants identified as Hindu (74.03%), followed by Muslims (12%), Christians (8.87%), and others (5.1%). The economic profile, based on MPCE, was relatively evenly distributed across quintiles, with the highest percentage in the poorer (21.1%) and poorest (20.96%) categories. Notably, a large majority (93.16%) reported participating in social activities. In terms of health indicators, 75.73% rated their health as good. However, 42.61% reported having limitations in IADL. Finally, 6.96% of respondents reported experiencing symptoms of depression.

**Table 1:** Socio-demographic characteristics of the study sample

Variables	Percentage (%)	Sample (n) Total *n* = 25,650
Age		
60–69	64.49	16,542
70–80	35.51	9108
Sex		
Male	46.44	11,912
Female	53.56	13,738
Marital Status		
Currently in union	66.72	17,114
Not in union	33.28	8536
Living arrangements		
Living alone	5.08	1163
Living with spouse and/or others	20.77	5154
Living with spouse and children	43.13	11,650
Living with children and others	27.12	6756
Living with others only	3.90	927
Caste		
Schedule caste	16.93	4342
Schedule tribe	15.94	4088
Other backward class	38.76	9943
Others	25.21	6467
Religion		
Hindu	74.03	18,989
Muslim	12.00	3,079
Christian	8.87	2,274
Others	5.10	1,308
Year of schooling		
0	54.17	13,893
1–5	18.80	4,821
6–9	12.90	3,310
10 +	14.13	3,625
Place of residence		
Rural	67.24	17,247
Urban	32.76	8,403
Social participation		
No participation	6.76	1,733
Having participation	93.16	23,896
Region		
North	18.81	4,826
Central	13.61	3,491
East	17.11	4,389
North-east	9.31	2,387
West	13.35	3,423
South	27.81	7,134
MPCE quintile		
Poorest	20.96	5,376
Poorer	21.10	5,412
Middle	20.39	5,230
Richer	19.44	4,987
Richest	18.11	4,645
Currently working status		
Currently not working	40.54	10,398
Currently working	31.03	7958
Self-rated health		
Good	75.73	19,421
Poor	24.27	6,225
IADL limitation		
No IADL limitation	57.38	14,717
Having IADL limitation	42.61	10,929
Depression		
No	93.04	23,866
Yes	6.96	1,784

Note: Due to missing data for some covariates, the number of observations varies across variables

### Life satisfaction levels by different socio-demographic characteristics of the study sample

Table 2 presents the bivariate difference in life satisfaction and various socio-demographic and health-related characteristics among older adults using the chi-square test. Significant differences (*p* < 0.001) in life satisfaction levels were observed across most variables. Older adults who are involved in grandchild care reported higher life satisfaction (51.35%) than non-caregivers (44.42%). Those older adults who are not in a union and living alone reported lower levels of life satisfaction. Higher life satisfaction is observed in those living with a spouse and children, with higher education levels, urban residents, and from other backward classes and caste groups. A strong gradient is observed with education—65.82% of those with 10 + years of schooling reported high life satisfaction. Life satisfaction is also higher among individuals from wealthier MPCE quintiles, those with social participation, good self-rated health, and no IADL limitations. Conversely, individuals reporting depressive symptoms had significantly lower levels of life satisfaction, with nearly half (48.83%) falling into the low satisfaction category. Regional and religious differences were also evident, with the western region showing the highest levels of high life satisfaction (69.09%).

**Table 2:** Bivariate difference in life satisfaction among older adults by socio-demographic characteristics

Variables	Level of life satisfaction			*p*-value
	Low	Medium	High	
Grandchild care				< 0.001
Yes	26.91	21.75	51.35	
No	32.79	22.79	44.42	
Age				< 0.366
60–69	31.23	22.89	45.88	
70–80	32.31	22.06	45.63	
Sex				< 0.001
Male	29.75	22.83	47.42	
Female	33.22	22.38	44.40	
Marital Status				< 0.001
Currently in union	29.46	23.33	47.21	
Not in union	35.61	21.22	43.17	
Living arrangements				
Living alone	42.30	20.72	36.96	< 0.001
Living with spouse and/or others	30.19	23.19	46.62	
Living with spouse and children	27.55	24.57	47.88	
Living with children and others	31.53	24.57	43.90	
Living with others only	35.92	23.19	40.88	
Caste				< 0.001
Schedule caste	39.49	22.70	37.80	
Schedule tribe	36.80	23.25	39.95	
Other backward class	30.55	22.43	47.02	
Others	25.84	22.63	51.53	
Religion				< 0.001
Hindu	31.58	22.56	45.87	
Muslim	32.43	24.16	43.41	
Christian	37.12	19.20	43.68	
Others	25.92	21.17	52.91	
Year of schooling				< 0.001
0	37.29	23.84	38.86	
1–5	29.87	22.67	47.46	
6–9	24.88	21.08	54.03	
10 +	15.73	18.45	65.82	
Place of residence				< 0.001
Rural	33.69	23.73	42.58	
Urban	26.30	19.65	54.05	
Social participation				< 0.001
No participation	43.93	18.86	37.21	
Having participation	30.67	22.86	46.47	
Region				< 0.001
North	33.20	24.11	42.69	
Central	32.31	26.14	41.54	
East	36.21	26.14	37.65	
North-east	26.14	28.79	45.07	
West	16.00	14.91	69.09	
South	38.23	19.62	42.15	
MPCE quintile				< 0.001
Poorest	37.40	23.81	38.79	
Poorer	33.71	23.21	43.08	
Middle	29.92	23.36	46.73	
Richer	27.61	22.49	49.90	
Richest	27.82	19.24	52.94	
Currently working status				< 0.001
Currently not working	32.26	21.54	46.20	
Currently working	31.33	23.72	44.94	
Self-rated health				< 0.001
Good	27.93	22.21	49.86	
Poor	42.40	23.66	33.93	
IADL limitation				< 0.001
No IADL limitation	28.79	22.19	49.03	
Having IADL limitation	34.89	23.05	42.06	
Depression				< 0.001
No	30.03	22.77	47.20	
Yes	48.83	20.66	30.51	

### The association between grandchild care and life satisfaction of older adults

Table 3 presents an ordered logistic regression analysis examining the association between caregiving for grandchildren and life satisfaction among older adults in India. The adjusted odds ratio for older individuals who reported providing care for their grandchildren exhibited significantly higher odds of life satisfaction than those who did not (OR = 1.16, CI: 1.10–1.23). This result indicates a consistent and statistically significant association between providing care for grandchildren and higher levels of life satisfaction among older adults, even after controlling a range of socio-demographic and health-related variables. Among socio-demographic variables, female respondents have higher odds of life satisfaction than males (OR = 1.10, CI: 1.03–1.17). Those not currently in a union had lower odds of life satisfaction than those in a union (OR = 0.77, CI: 0.61–0.96). Living arrangements significantly influenced life satisfaction. Compared to those living alone, higher odds were observed among those living with children and others (OR = 1.38, CI: 1.22–1.56). Caste differences were evident. Compared to Scheduled Castes, individuals from Other Backward Classes (OR = 1.26, 95% CI: 1.17–1.35) and from general (other) category having higher odds of life satisfaction (OR = 1.25, 95% CI: 1.16–1.35). Furthermore, as years of schooling increase, the odds of life satisfaction also increases. Social participation was positively linked to life satisfaction (OR = 1.12, CI: 1.02–1.23). As measured by the MPCE quintile, economic status demonstrated a graded positive association. Compared to the poorest group, the odds of life satisfaction increased with each higher quintile, from poorer (OR = 1.14, CI: 1.06–1.23) to richest (OR = 1.38, CI: 1.28–1.50). Additionally, currently working individuals had slightly lower odds of life satisfaction than those not working (OR = 0.92, CI: 0.87–0.98). In contrast, poor self-rated health (OR = 0.63, CI: 0.60–0.67), IADL limitations (OR = 0.88, CI: 0.84–0.93), and depression (OR = 0.54, CI: 0.49–0.59) were all significantly associated with lower odds of life satisfaction.

**Table 3:** Ordered logistic regression results showing the association between grandchild care and life satisfaction among older adults in India

Life satisfaction	Total	
	OR	95% CI
Grandchild care		
No ®		
Yes	1.16***	(1.10—1.23)
Age		
60 −69 ®		
70–80	1.06*	(1.00—1.11)
Sex		
Male ®		
Female	1.10**	(1.03—1.17)
Marital Status		
Currently in union ®		
Not in union	0.77*	(0.61—0.96)
Living arrangements		
Living alone ®		
Living with spouse and/or others	1.08	(0.85–1.39)
Living with spouse and children	1.15	(0.90–1.47)
Living with children and others	1.38***	(1.22–1.56)
Living with others only	1.22*	(1.03–1.44)
Caste		
Schedule caste ®		
Schedule tribe	1.06	(0.97—1.16)
Other backward class	1.26***	(1.17—1.35)
Others	1.25***	(1.16—1.35)
Religion		
Hindu ®		
Muslim	0.85***	(0.79—0.92)
Christian	1.11*	(1.01—1.23)
Others	1.14*	(1.01- 1.27)
Year of schooling		
0 ®		
1—5	1.29***	(1.20—1.37)
6—9	1.41***	(1.30- 1.52)
10 +	2.06***	(1.89—2.24)
Place of residence		
Rural ®		
Urban	1.13***	(1.06—1.19)
Social participation		
No participation ®		
Having participation	1.12*	(1.02—1.23)
Region		
North ®		
Central	0.92*	(0.85—1.01)
East	0.70***	(0.65—0.77)
North-east	0.90*	(0.81—0.99)
West	2.14***	(1.96—2.36)
South	0.78***	(0.73—0.86)
MPCE quintile		
Poorest ®		
Poorer	1.14***	(1.06—1.23)
Middle	1.22***	(1.13—1.31)
Richer	1.28***	(1.18- 1.38)
Richest	1.38***	(1.28—1.50)
Currently working status		
Currently not working ®		
Currently working	0.92**	(0.87—0.98)
Self-rated health		
Good ®		
Poor	0.63***	(0.60—0.67)
IADL limitation		
No IADL limitation ®		
Having IADL limitation	0.88***	(0.84—0.93)
Depression		
No ®		
Yes	0.54***	(0.49—0.59)
/cut1	−0.33	(-0.62—-0.06)
/cut2	0.75	(0.48- 1.03)

^*^p < 0.05, **p < 0.01, ***p < *0.001; ®* = *Reference Category*

### PSM results

**Fig. 2:**
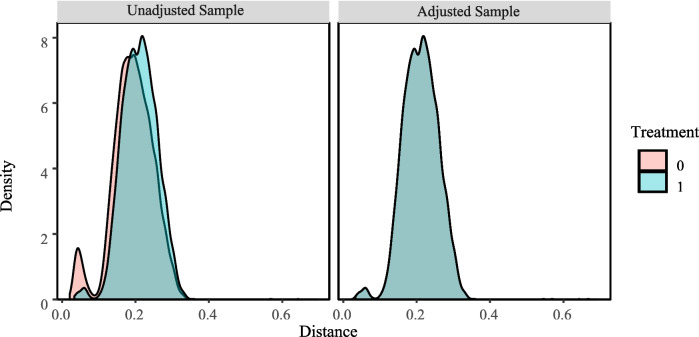
Balance distribution

**Fig. 3:**
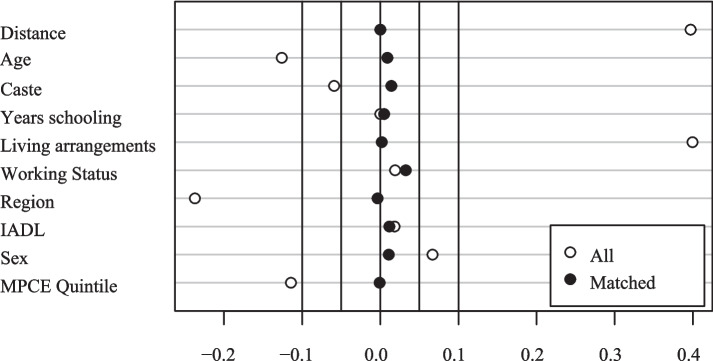
Standardized mean differences

This study further utilized PSM to validate whether life satisfaction was higher among older adults who provided grandchild care than those who did not after eliminating selective bias. This study utilized a nearest-neighbor matching methodology to calculate the average treatment effect. The distribution plots demonstrating the balance of the sample are presented in Fig. 2, while the standardized mean differences for both the matched and all data are illustrated in Fig. 3. Furthermore, Table 4 provides detailed information regarding the matched and unmatched cases.

**Table 4:** Details of matched and unmatched cases

Cases	Control	Treated
All	20,622	5028
Matched	5028	5028
Unmatched	15,594	0
Discarded	0	0

**Fig. 4:**
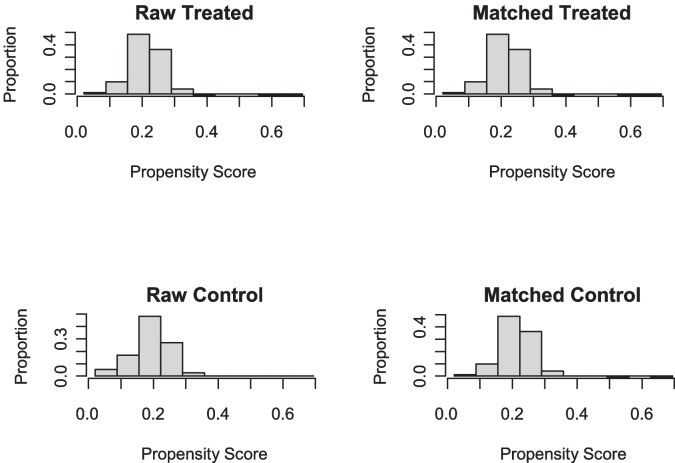
Histogram of Propensity Score

A summary of the balance for all data is delineated in Table 5. Table 6 shows the summary of the balance for matched data, and a strong matching effect is demonstrated. Figure 4 displays histograms that illustrate the propensity scores for the raw treated and matched treated groups alongside those of the raw control and matched control groups.

**Table 5:** Summary of balance for all data

Variables	Means Treated	Means Control	Std. Mean Diff
Distance	0.2113	0.1923	0.3971
Age	1.3083	1.3665	−0.1260
Caste	5.0833	5.9570	−0.0591
Years Schooling	1.8739	1.8738	0.0001
Living Status	1.9887	1.9464	0.3995
Working Status	0.8914	0.8760	0.0189
Region	3.2546	3.6568	−0.2373
IADL	0.4712	0.4343	0.0182
Sex	1.5623	1.5291	0.0668
MPCE Quintile	2.7987	2.9576	−0.1143

**Table 6:** Summary of balance for matched data

Variables	MEANS TREATED	MEANS CONTROL	STD. MEAN DIFF
Distance	0.2113	0.2112	0.0001
Age	1.3083	1.3041	0.0090
Caste	5.0833	4.8745	0.0141
Years Schooling	1.8739	1.8685	0.0049
Living Status	1.9887	1.9885	0.0019
Working Status	0.8914	0.8648	0.0326
Region	3.2546	3.2605	−0.0035
IADL	0.4712	0.4477	0.0116
Sex	1.5623	1.5569	0.0108
MPCE Quintile	2.7987	2.7999	−0.0009

Table 7 shows the average treatment effect of caring for grandchildren on the life satisfaction of older people is 0.057, indicating a statistically significant impact of caregiving on enhancing life satisfaction among older adults. This result is consistent with the previous logistic regression outcomes. Therefore, it can be concluded that the impact of caring for grandchildren on the life satisfaction of older individuals is robust and statistically significant, as evidenced by the strong matching effect and the observed average treatment effect.

**Table 7:** Average treatment effect

PSM	Grandparent caregiving	*p*	SE	Z
Nearest Neighbor matching method	0.057	< 0.001	0.0161	3.53

## Discussions

Using a nationally representative dataset, this study examined the association between grandchild caregiving and life satisfaction among older adults in India. The findings reveal that grandchild caregiving is positively associated with higher life satisfaction. Older adults who provided care for grandchildren had 16% higher odds of reporting better life satisfaction than non-caregivers, even after adjusting for a wide range of socio-demographic and health-related variables. This supports the Role Enhancement Theory, which states that possessing multiple roles affords individuals opportunities for social interaction, personal growth, and accomplishment [27]. The extra role of grandchild caregivers could inject new meaning into life and eliminate the sense of role deprivation related to retirement, making older adults' lives more fulfilling and positive [25]. This finding is also consistent with the previous studies, which explored the association between grandchild care and life satisfaction [4, 29]. Caring for grandchildren can allow their children to concentrate on their careers or pursue other personal goals with greater latitude [3]. As a natural response, adult children may feel motivated to provide increased financial and instrumental support to their parents, strengthening their relationships and contributing to their life satisfaction [32]. Furthermore, our study also revealed that the correlation between grandchild care and life satisfaction significantly varied based on age, gender, residence, living arrangement, self-rated health, IADL Limitation, education attainment, marital status, and participation in social activities. The older age group demonstrated higher life satisfaction than those relatively young. One possible explanation could be that the oldest adults in India have more time to devote to family, derive fulfillment from nurturing family bonds, uphold cultural values that place high importance on family connections, and prioritize relationships as they age. Notably, older women reported significantly higher life satisfaction than men. This may be attributed to women's stronger social bonds, greater emotional investment in family roles, and higher involvement in caregiving. Cultural norms in India often position women as primary caregivers, and their engagement in grandchild care may be more emotionally meaningful and socially reinforced than for men [1]. Education emerged as a strong predictor, with higher educational attainment significantly increasing life satisfaction—likely due to better coping skills, greater social engagement, and economic security. Similarly, economic well-being, measured via MPCE quintiles, demonstrated a dose–response relationship with life satisfaction, consistent with the established link between material resources and psychological well-being in old age. Social participation and urban residence were also positively associated with life satisfaction, suggesting that access to social networks and urban amenities may enhance older adults' quality of life. In contrast, poor self-rated health, functional limitations (IADL), and depression were strongly associated with lower life satisfaction, reinforcing the importance of physical and mental health in ageing outcomes.

The findings of this study offer a meaningful theoretical contribution to the literature on ageing in the Indian context. The positive association between grandchild caregiving and life satisfaction among older adults supports the role enhancement theory, which posits that engaging in meaningful social roles—such as caregiving—can enhance an individual’s sense of purpose and overall well-being. This suggests that, within the socio-cultural fabric of India, assuming caregiving responsibilities may serve as a valued role that enriches life satisfaction in later years.

### Limitations of the study

The study focused solely on the relationship between caring for grandchildren and the life satisfaction of older adults. It didn't consider how much or how often the grandparents provided care. Future research could examine how the intensity of caregiving impacts older adults' life satisfaction for a more complete picture. The data on life satisfaction were collected on a self-reported questionnaire. Therefore, some recall bias may be introduced.

This study focused on India, so the findings might not apply to other cultures. In India, strong family bonds might explain why caring for grandkids gives grandparents more positive life satisfaction. More research is needed to see if this happens in other places.

## Conclusion

Our study aimed to explore the relationship between grandchild caregiving and the life satisfaction of elderly individuals in India, and the results revealed that grandparenting positively impacts the happiness and well-being of older adults. Participating in grandchild caregiving is crucial for enhancing overall life satisfaction among older adults. These findings have important implications for policymakers, healthcare professionals, and families. Policymakers and healthcare providers need to recognize and support the value of grandchild care in improving the quality of life for older individuals. Implementing interventions to strengthen family support systems and providing resources for caregiving can make grandparenting relationships even more beneficial for the happiness and well-being of older adults in India. By strengthening these family ties, we can create an age-friendly society where older adults feel valued and supported and enjoy a fulfilling and satisfying later life.

## Data Availability

The study is based on secondary data source, is freely available in the public domain through https://www.iipsindia.ac.in/content/LASI-data.

## Abbreviations

aOR: Adjusted Odd Ratio
CI: Confidence Interval
CIDI-SF: Short Form Composite International Diagnostic Interview
IADL: Instrumental Activities of Daily Living
LASI: Longitudinal Ageing Study in India
MPCE: Monthly Per Capita Consumption Expenditure
OR: Odd Ratio
PSM: Propensity Score Matching
SWLS: Satisfaction with Life Scale
